# Role of CT in Assessment of Unresectable Wilms' Tumor Response after Preoperative Chemotherapy in Pediatrics

**DOI:** 10.1100/tsw.2008.96

**Published:** 2008-07-13

**Authors:** Huda Refaie, Mohamed Sarhan, Ashraf Hafez

**Affiliations:** Mansoura University-Elgomhoria st, Mansoura, 35516, Egypt

**Keywords:** CT, Wilms' tumor, chemotherapy, unresectable

## Abstract

The purpose of this study was to define the structural response of unresectable Wilms' tumor following preoperative chemotherapy by computed tomography (CT). We also compared CT changes in relation to histopathological nature. The study included 36 patients with 41 nephroblastomas. All were examined by CT before preoperative chemotherapy using multiphasic CT protocol study. The unresectability was diagnosed by CT imaging. All patients were subjected to fine-needle biopsy (FNB) to confirm the diagnosis and to define the histopathological type before preoperative chemotherapy. Five patients had unfavorable pathology and 31 patients with 36 nephroblastomas had favorable pathology. All patients received first line of treatment. Follow-up of these patients by CT at the 6th week was reviewed. All of our patients were diagnosed as unresectable Wilms' tumor by CT. Preoperative chemotherapy was started. Among our patients, 28 (77.8%) gave good response in the form of significant reduction in tumor size, disappearance of one tumor in two cases with bilateral WT and inferior vena cava (IVC) thrombus, and increased nonenhancing necrotizing content. Two patients with unfavorable pathology did not show any response. The remaining six patients gave partial response. CT can be used to evaluate tumor response and resectability after preoperative chemotherapy with high accuracy. The response to preoperative chemotherapy is not related to the histopathological classifications.

## INTRODUCTION

Wilms' tumor (WT) is the fifth most common pediatric malignancy and the most common renal tumor in children. Treatment is a living example of success achieved through a multidisciplinary collaboration of the National Wilms' Tumor Study Group (NWTSG) and the Societe Internationale d'Oncologie Pediatrique (SIOP). Due to the cooperative efforts of oncologists, surgeons, and pathologists, and with the introduction of chemotherapy with vincristine, dactinomycin (actinomycin D), and doxorubicin, the overall survival (OS) rate has risen to 90% in the last 30 years[1].

The North American protocol recommends primary surgical excision of nephroblastoma. Inoperable WTs constitute about 28% (stage III = 23% and stage V = 5%) of total cases of WT. The inoperable criteria most commonly utilized are huge size of the tumor, involvement of adjacent vital structures, intracaval involvement above the level of the hepatic veins, and atrial tumor extension. These factors significantly increase the risk of surgical morbidity, principally hemorrhage, during initial nephrectomy[2].

Preoperative chemotherapy has been found to almost always ease surgical resection by reducing tumor size, decreasing the incidence of tumor rupture and spoilage by 50%, and if inferior vena cava (IVC) thrombus is also present, preoperative chemotherapy will reduce the cavotomy rate by 50%[3]. Marked improvements in the postoperative histology in favorable-histology WT patients compared with those who did not receive preoperative chemotherapy[4] are shown in Table 1.

**TABLE 1**
**Histological Subtypes of WT in Immediate Surgery vs. Preoperative Chemotherapy**

| Histology Subtype | Immediate Surgery | Preoperative Chemotherapy |
|---|---|---|
| Epithelial predominant | 15.5% | 3.1% |
| Stromal predominant | 0 | 14.0% |
| Blastemal predominant | 39.4% | 9.3% |
| Mixed | 45.1% | 29.4% |
| Regressive predominant | 0 | 37.6% |
| Completely necrotic | 0 | 6.6% |

WTs are classified into two major types, according to the American Cancer Society, depending on their histology.

1. Favorable histology — These WTs have a favorable appearance under the microscope. While the cells are not quite normal looking, there is no anaplasia. The chance of cure for these tumors is very good.
2. Unfavorable histology (anaplastic WT) — These WTs have an unfavorable appearance under the microscope. The look of the cancer cells varies widely, and the nuclei (the central part of the cell that contains the DNA) of the cells tend to be very large and distorted. This is called anaplasia. The more anaplasia that is found, the poorer the chance for a cure[5].

On the National Wilms' Tumor Study-5 (NWTS-5), inoperable patients were treated after biopsy by initial chemotherapy with vincristine and dactinomycin, with or without doxorubicin[6,7,8]. If no reduction in tumor size occurred after using three drugs, then radiation therapy was used[9]. Surgery was performed as soon as sufficient tumor shrinkage occurred, generally at week 6 of therapy. Because of the 5–10% error rate in preoperative diagnosis of renal masses after radiographic assessment, confirmation of the diagnosis by biopsy (which may be performed percutaneously) was obtained prior to chemotherapy[7,10].

By repeated cross-sectional modalities in cancer patients, large amounts of data can be acquired. Currently, spiral CT can monitor tumor treatment response by tumor diameter change and subjective features: estimation of the tumor size by identifying the largest extension at axial slice measure (long axis, LA); the diameter perpendicular to LA, on the same slice measure (short axis, SA); the most cranial and the most caudal slice showing craniocaudal diameter.

The aim of this work was to define the structural response of unresectable WT following preoperative chemotherapy by CT compared to tumor histopathological class.

## PATIENTS AND METHODS

The study included 36 patients with 41 unresectable nephroblastomas. Patients were managed at the Urology and Nephrology Center of Mansoura University during the period from June 2004 to January 2006. Patients' median age was 4 years (range: 16 months to 7 years). Male to female ratio was 1:1.1. All were subjected to abdominal CT examination before receiving chemotherapy and completed with CT chest. Patients were assigned a pretreatment stage: stage III (31 patients) and stage V (five patients) (Table 2).

**TABLE 2**
**Clinical and Diagnostic Features of the Studied Cases**

| Variables | Stage III No. = 31 | Bilateral WT No. = 5 | Total No. = 36 |
|---|---|---|---|
| Age (years): | | | |
| Median, range | 6, 4–7 | 2.5, 1.3–4 | 4, 1.3–7 |
| Sex (M:F) | 16:15 (1.01:1) | 1:4 (1:4) | 17: 19 (1:1.1) |
| Tumor dimensions in CT: | | | |
| Median (cm) | 12 × 9 × 8 | 8 × 7 × 6 | 10 × 8 × 7.5 |
| Histology: | | | |
| Favorable | 26 (83.9%) | 5 (100%) | 31 (86.1%) |
| Unfavorable | 5 (16.1%) | 0 (0%) | 5 (13.9%) |
| Preoperative chemotherapy: | | | |
| VCR+AMD | 8 (25.8%) | 0 (0%) | 8 (22.2%) |
| VCR+AMD+DOX | 17 (54.8%) | 5 (100%) | 22 (61.1%) |
| Other | 6 (19.4%) | 0 (0%) | 6 (16.7%) |

The unresectability due to bilaterality (n = 5), adherence to adjacent organs (n = 26), and IVC thrombus (n = 5) was diagnosed by CT imaging. Fine-needle biopsy (FNB) was done to define the histopathological class. Thirty-one patients had favorable and five had unfavorable pathology. The chemotherapy regimen included dactinomycin and vincristine (eight patients); dactinomycin, vincristine, and doxorubicin (22 patients); and other (six patients).

Abdominal CT examination at the 6th week after receiving chemotherapy was done to evaluate the response. Timed follow-up CT settings over the study period were done to confirm remission.

The study was carried at 4 raw multidetector CT scanner, Light speed Plus(General Electric Medical System, Milwaukee, WI, USA).We did a precontrast scan of the abdomen from the level of diaphragmatic copula down to the iliac crest at 5-mm slice thickness, then we repeated the scan for the chest and abdomen after injection of 2-ml/kg nonionic contrast media iopamidol (Iopamiro 300, Bracco, Milan, Italy) using an automatic pump.

## RESULTS

Twenty-eight patients (77.8%) gave good response in the form of significant reduction in tumor size from median dimensions of 10 × 8 × 7.5 cm to 7 × 6 × 4 cm ($p$ = 0.03). Other signs of good response included disappearance of one tumor in two cases with bilateral WT and IVC thrombus (n = 3), and increased nonenhancing necrotizing area (n = 22) (Table 3) (Figs. 1, 2, and 3).

**TABLE 3**
**Response to Preoperative Chemotherapy as Evaluated by CT**

| Variables | Stage III No. = 31 | Bilateral WT No. = 5 | Total No. = 36 |
|---|---|---|---|
| Tumor size reduction | 23 (74.2%) | 5 (100%) | 28 (77.8%) |
| Disappearance of IVC thrombus | 3 (9.7%) | 0 (0%) | 3 (8.3%) |
| Disappearance of tumor | 0 (0%) | 2 (40%) | 2 (5.6%) |
| Increased necrotic areas | 18 (58.1%) | 4 (80%) | 22 (61.1%) |

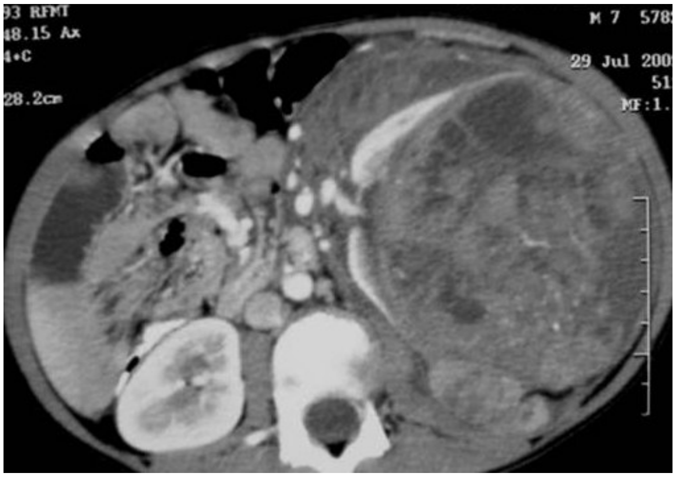

A

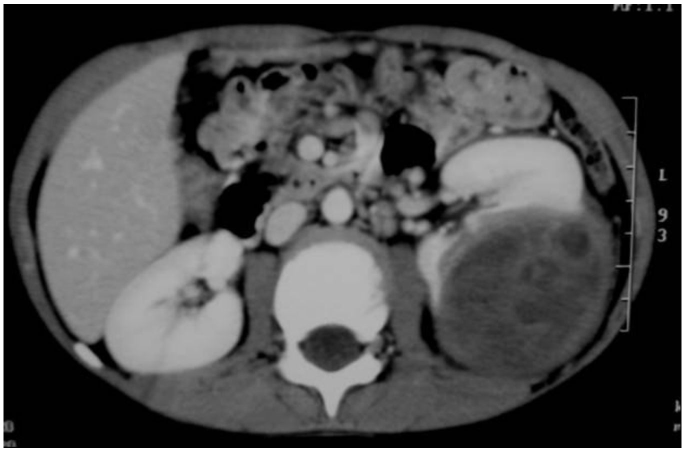

B

**FIGURE 1.** Postcontrast axial CT scan of the kidneys in a 5-year-old male shows (A) prechemotherapy, there is a large mass replacing the left kidney with perirenal extension; (B) postchemotherapy, there is marked reduction in the size of the mass.

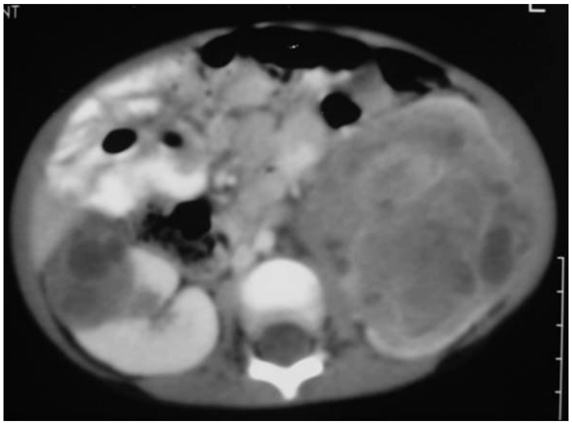

A

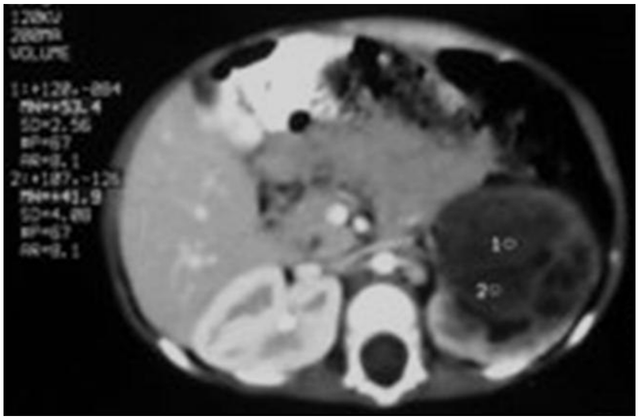

B

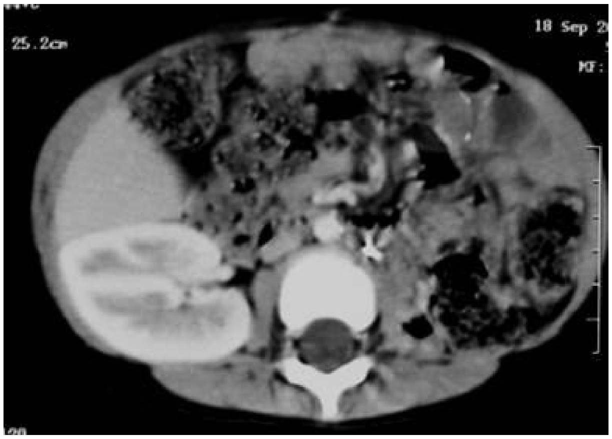

C

**FIGURE 2.** Postcontrast axial CT scan of the kidneys in a 4-year-old girl shows (A) prechemotherapy scan, bilateral renal masses with small right one and large mass at the left side extending into the perirenal space; (B) postchemotherapy scan, there is complete resolution of the right renal mass with significant reduction in the size of the left one; (C) post–left nephrectomy with free right kidney and no abnormalities at the left renal bed.

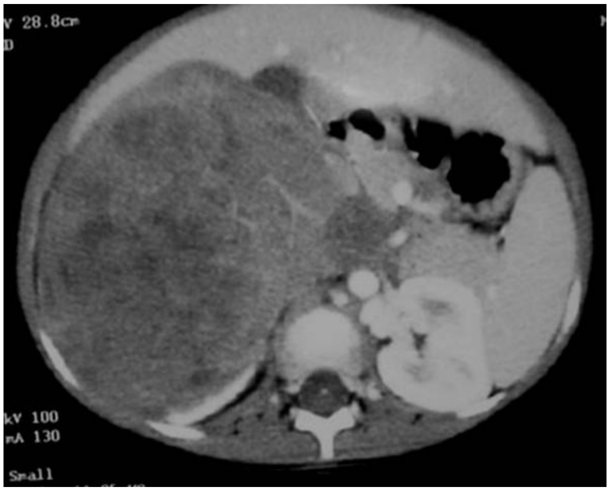

**A**

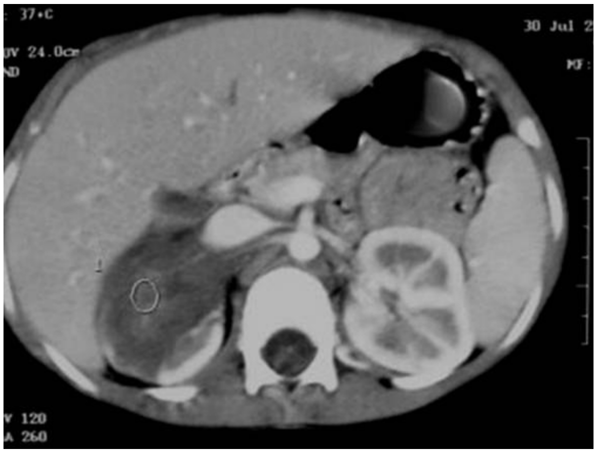

**B**

**FIGURE 3.** CT findings in a 6-year-old girl with right WT. (A) Preoperative scan shows a large renal mass with nonopacified IVC; (B) 6 weeks after chemotherapy, there is marked reduction in the size of the mass with resolution of the IVC thrombus.

Two patients with unfavorable pathology did not show any response to therapy and surgical excision was done, followed by a postoperative combination of chemotherapy and radiotherapy and second-look surgery. On the other hand, six patients with favorable pathology showed some response and needed intensive therapy in addition. The intensification of preoperative chemotherapy using ifosfamide and etoposide rendered them resectable on following CT evaluation after another 6 weeks.

Surgery was done to all patients at the Urology and Nephrology Center of Mansoura University. Median time to do nephrectomy was 68.5 days from diagnosis.

Histopathological evaluation of the resected tumors confirmed the good response to the preoperative chemotherapy. Massive necrosis, hemorrhage, and even absence of any tumor cells in the postoperative pathological study were noted.

Radiological and postoperative histopathological studies did not differ significantly between favorable and unfavorable groups. Under-staging was noticed in 20 cases of preoperative stage III cases

(64.5%) where the new stage was stage I in eight cases and II in 12 cases. The difference between postoperative histopathological staging and postchemotherapy CT staging is presented in Table 4.

**TABLE 4**
**Postchemotherapy CT Staging vs. Postoperative Histopathological Staging**

| Histopatological Stage | CT Stage | | | |
|---|---|---|---|---|
| | I | II | III | IV |
| I | 8 | 8 | 4 | 2 |
| II | 0 | 4 | 2 | 2 |
| III | 0 | 0 | 5 | 1 |
| Total | 8 | 12 | 11 | 5 |

Postoperative chemotherapy was given according to the preoperative stage III and included dactinomycin, vincristine, and doxorubicin for 24 weeks. Stage V cases were dealt with also according to the prechemotherapy stage where three patients received dactinomycin, vincristine, and doxorubicin for 24 weeks and two received dactinomycin and vincristine for 24 weeks.

## DISCUSSION

Computerized tomography (CT) is performed in most children with renal masses, and provides helpful anatomical and functional data. CT with and without intravascular contrast material can detect sharp demarcations between tumor and normal renal parenchyma, and assess the relative function of the contralateral kidney. Because the entire abdomen is imaged with CT, there was initially great enthusiasm for its ability to stage cases accurately preoperatively, but with further experience, much of this initial promise has waned[11].

CT can suggest tumor extension into perirenal fat and adjacent organs, but only exploration can confirm it. For right tumors, CT has a high false-positive rate for hepatic invasion, but it is 100% accurate when invasion is excluded. Although CT can detect retroperitoneal adenopathy, benign nodal enlargement is commonly seen in children and, therefore, adenopathy, even palpated at exploration, does not correlate well with metastatic disease. CT is also of limited accuracy in excluding vascular invasion[12].

We reviewed 36 children enrolled in the present study who received preoperative treatment for tumors unable to be resected at surgery or judged inoperable by imaging evaluation. Patients' median age was 4 years, with a male to female ratio of 1:1.1, which resembles a study by Hung et al.[13] who reported a mean age of 3.7 years at diagnosis and M/F ratio of 1.04. Most bilateral tumors occurred in females and this agrees with Hung et al., where all bilateral tumors occurred in females.

Preoperative biopsies were performed on all patients by FNB prior to chemotherapy. Anaplastic histology was present in 13.9% of the studied cases and this was double the finding in Hung et al., with just 6.1% anaplastic histology. Patients were assigned a pretreatment stage: stage III (31 patients) and stage V (five patients). The chemotherapy regimen included dactinomycin and vincristine (eight patients); dactinomycin, vincristine, and doxorubicin (22 patients); and other (six patients). Response to therapy was assessed after the 6th week of chemotherapy. Good response was noticed in 77.8% of our patients, which was near to that described by Ritchey et al.[14], who noted partial responses in 85% of their patients.

Poor response was seen in 22.2% of cases, while Ritchey et al. showed a 10% failure of response. However, 75% of the initial nonresponders were rendered responders after using a more intensive regimen. There were no significant differences in preoperative response to the different chemotherapy regimens. The median time interval from diagnosis to nephrectomy was 68.5 days vs. 58.5 days, and nephrectomy was completed in all patients after preoperative treatment vs. 93% seen in Ritchey et al.

Postoperative histopathological evaluation of the tumors showed that the CT scan appeared to have poor correlation to histological staging and this was matched with the results of Gow et al.[15]. However, CT is still preferred and widely accepted as the patient will always require some additional cross-sectional imaging anyway. CT is more readily available, quicker, and easier to perform than MRI in small children (the increased speed reducing the need for sedation) and this was also the recommendation of Maudgil and McHugh[11].

## CONCLUSION

The use of preoperative treatment can facilitate subsequent surgical resection in selected patients with inoperable WT, although some of these large tumors have a somewhat poor initial response. However, nephrectomy was completed in all patients after preoperative treatment. Also, preoperative treatment will lead to less accurate surgical and pathologic staging, and undertreatment should be avoided in these high-risk patients.

CT can be used to evaluate tumor response after preoperative chemotherapy with high accuracy and its resectability. The response to preoperative chemotherapy is not related to the histopathological class.
